# Comparative effectiveness and safety of direct-acting oral anticoagulants (DOACS) for the reduction of recurrent venous thromboembolism in cancer patients: A protocol for systematic review and network meta-analysis using a generalized pairwise modeling methodology

**DOI:** 10.1097/MD.0000000000019679

**Published:** 2020-04-03

**Authors:** Mohammed ibn-Mas’ud Danjuma, Mouhand F.H. Mohamed, Mohamad Nabil ElShafei, Haajra Fatima, Shaikha Al Shokri, Sara Mohamed, Ibrahim Yusuf Abubeker, Anand Kartha, Abdel-Naser Elzouki, Mohamed Gaafar Hussein Mohamedali, Yahya Mahgboub, Mubarak Bidmos

**Affiliations:** a Qatar University, College of Medicine, QU, Health, Doha; b Internal Medicine Department, Hamad General hospital, Hamad Medical Corporation, Qatar; c Rochester regional health Unity Internal Medicine Department; d Wirral University Hospital, NHS foundation Trust, Wirral, Liverpool, UK.

**Keywords:** cancer associated thrombosis, cancer thrombosis, DOAC, DVT, NOAC, novel oral anticoagulant, pulmonary embolism

## Abstract

**Background:**

There has been a significant improvement in both our understanding and therapeutic choices available to clinicians for the management of cancer associated thrombosis (CAT). Even with the recent publication of a systematic review and landmark trials demonstrating the non-inferiority of DOACS-based anticoagulation strategy compared to the standard of care in patients with CAT, there is unresolved uncertainty regarding the exact hierarchy of risks and effectiveness of various DOAC analogues in these cohorts of patients.

**Method:**

We will carry out a network meta-analyses, utilizing a novel generalized pairwise methodology to generate direct and indirect comparisons between the various DOAC analogues. We will search the following databases for studies that satisfies pre-specified inclusions criteria; these include PubMed, EMBASE, Cochrane library, Clinicaltrials.gov, conference abstracts among other sources. The primary efficacy and safety outcomes are recurrent VTE and major hemorrhagic events, respectively. Two reviewers will Search the databases independently with the view to identify studies that meet eligibility criteria. The methodological quality of the included studies will be determined using a recently validated risk of bias assessment tool.

**Results:**

We expect that the result of this review will ascertain the hierarchy of risks and effectiveness of various DOAC analogues in patients with CAT.

**Conclusion:**

Results of this review will assist in informed decisions making regarding therapeutic guidelines of DOAC in CAT.

## 1 Introduction

Cancer associated thrombosis is a well-recognized and increasing morbidity in cancer patients both on and off chemotherapy.^[1,2]^ The usual standard of care in patients with CAT has been the use of low molecular weight heparin (LMWH) for various duration depending on the site of thrombosis (either Pulmonary embolism [PE] or deep venous thrombosis [DVT]).^[3]^ LMWH in particular has been shown to be non-inferior and have a more tolerable pharmacokinetics in patients with CAT compared to vitamin K antagonists (VKA).^[3,4]^The issues associated with the strategy of anticoagulation with VKA have been exhaustively discussed elsewhere;^[1,3–5]^ some of this however, includes labile INR, risk of drug-drug interactions with chemotherapeutic agents as well as the need for regular INR monitoring among others^[1,4,5]^ Since the demonstration of the non-inferiority of Direct Oral Anticoagulants (DOAC) as a strategy for anticoagulation (compared to usual standard of care) in various clinical risks including CAT, there has been a growing interest in the comparative effectiveness and risks of the various analogues within the DOAC family.^[2,6–10]^ It is of therapeutic importance to ascertain the exact league of effectiveness of these agents as this would assist enormously in informing oncologists, hematologists and internists therapeutic decisions and therapeutic commissioning. There have been recent attempts at a comparative synthesis of the current data regarding DOACs and other anticoagulant classes in patients with CAT,^[6,11]^ however, none have attempted a direct and indirect head to head pairwise comparison between the various DOAC analogues.

In this review we intend to carryout a direct and an indirect comparison of efficacy and safety between the various DOAC analogues utilizing a novel meta-analytical methodology (Generalized pairwise modeling).

## 2 Objectives

The main objective is to determine the exact league of effectiveness and safety between the various DOAC in patients with CAT. This will be attempted by direct and indirect comparison via a network meta-analysis of studies exploring the efficacy and safety of various DOAC analogues.

## 3 Overview

We propose to conduct this review in strict adherence to the Preferred Reporting Items for Systematic Reviews and Meta-Analyses guidelines (PRISMA).^[12]^ The review protocol is registered with the International Prospective Register of Systematic Reviews (registration number: CRD42019154464).

## 4 Methodology

### 4.1 Population

Studies evaluating various DOAC analogues against standard of care in patients with CAT. We will accept randomized controlled trials and observational studies with low risk of bias.

### 4.2 Intervention

DOAC analogue.

### 4.3 Comparison

Usual standard of care or placebo.

### 4.4 Study outcomes

The primary efficacy outcome is the recurrence of VTE events, whilst the primary safety outcome is the rate of major bleeding.

## 5 Study eligibility criteria

### 5.1 Inclusion criteria

1. Age >18 years with cancer associated thrombosis
2. Randomized controlled trials, observational studies.
3. Patients on at least one DOAC intervention group in comparison with other anticoagulants (LMWH and VKA) or placebo.
4. Studies with at least one efficacy or safety outcome (recurrent VTE and bleeding episodes).
5. Studies published in English language.
6. Studies that fulfill rigorous bias risk assessment

### 5.2 Exclusion criteria

We will exclude all other studies that fail to meet the aforementioned inclusion criteria.

### 5.3 Literature sources

A comprehensive search of the following databases will be attempted:

PubMed, MEDLINE, EMBASE, Clinicaltrials.gov, Cochrane Controlled Trials Register (CCTR), Cochrane Database of Systematic Reviews (CDSR), Database of Abstracts of Reviews of Effectiveness (DARE), Health Technology Assessment Database (HTA), Index to Scientific and Technical proceedings (ISTP), National Research Register (NRR), NHS Economic Evaluation Database (NHS EED) to identify relevant studies.

### 5.4 Search strategy

We will restrict ourselves to studies published in English language only. No date restriction will be applied. Example of a search strategy is the following; ((((((((((pulmonary thromboembolism[MeSH Terms]) OR (thromboembolism[MeSH Terms])) OR (thromboembolism, venous[MeSH Terms])) OR (thrombosis)) OR (VTE)) OR (pulmonary embolism)) OR (Deep venous thrombosis)) OR (DVT)) OR (venous thromboembolism)) AND ((((((((malignancy) OR (cancer)) OR (lung ca)) OR (oncology)) OR (malignant)) OR (cancerous)) OR (hematologic malignancy[MeSH Terms])) OR (hematologic malignancies[MeSH Terms]))) AND ((((((((((doacs) OR (NOACS)) OR (Rivaroxaban)) OR (Edoxaban)) OR (apixaban)) OR (Dabigatran)) OR (novel oral anticoagulant)) OR (novel oral anticoagulants)) OR (Direct oral anticoagulant)) OR (Direct oral anticoagulants)).

### 5.5 Criteria for study selection

Two reviewers (MID) and (MFHM) will independently assess the titles and abstracts of the articles retrieved via database search using the inclusion criteria as a guide. Secondly, full text of eligible articles will be retrieved and assessed for eligibility. If disagreement ensues between reviewers a discussion will happen to settle the disagreement. However, if the disagreement is not settled by discussion, a third reviewer (MAB) will settle the disagreement guided by the protocol.

### 5.6 Data collection and abstraction

An excel data collection sheets will be developed for the purpose of data acquisition, 2 reviewers (MID & MFHM) will extract data from selected studies using these sheets. The variables of interest include; the study ID, year of publication, first author name, study design, study sample size, index cancer definition, cancer sites, DOAC dosing, study outcomes definition, journal, intervention, comparator, number of patients with and without the events of interest, follow-up duration, and effect sizes of both primary and safety outcome. The first authors last name and the year of publication will be used to identify all studies within the excel specific database.

### 5.7 Study quality and risk of bias assessment

We will assess the quality and risk of bias of studies included in our systematic review using the novel risk of assessment checklist and framework published by Stone et al.^[13]^ The checklist has 36 questions that incorporates the Cochrane checklist,^[14]^ We will consider the randomization of RCT adequate if 1 of the following concealment strategies were present; computer sequence generation, web-based sequence generation or opaque sealed envelopes. Blinding will be considered adequate if the participants were not able to guess which groups they are allocated to. We will consider attempts of limiting information bias adequate if care was delivered equally to both groups, with limited comparable co-interventions and consistent objective outcomes. The full checklist and its domains can be found in Supplemental Digital Content (Appendix 1). Reviewers (MID & MFHM) will independently score individual studies against all 36 checklist domains and questions. The overall score will be used to determine the quality and risk of bias of each study. Among the nine domains of the checklist we will report the most significant source of bias in each study. The third reviewer (MAB) will settle disagreement, not settled via discussion, arising between the study two reviewers (MID & MFHM).

## 6 Data analyses and synthesis

### 6.1 Estimation and calculation of effect sizes

For the primary efficacy and safety outcomes (VTE recurrence and bleeding events rates) all initial analyses will be attempted using pooled effect sizes (OR, RR, HR) reported from selected studies. If data on Effect sizes are not available, it will be requested from the study authors (including the relevant confidence intervals). Secondary outcomes will be calculated using the same procedure as for primary outcome.

### 6.2 Pooled estimates for change in study outcomes

A network maps will be drawn to illustrate the interventions that are directly compared against each other, including the magnitude of evidence available for each treatment and its comparator.

### 6.3 Meta-biases evaluation

Study publication bias and other small study effect will be determined will be evaluated using comparison adjusted funnel and Doi plots.^[15]^

### 6.4 Analytical software for data synthesis

MetaXL software will be used for all statistical analysis. (version 5.3 © EpiGear International Pty Ltd ABN 51 134 897 411 Sunrise Beach, Queensland, Australia, 2011–2016).

### 6.5 Strategy for dealing with missing data

In the event of missing data, we will contact the corresponding authors of the primary studies involved to provide us with the missing data. Where this is not possible then we will attempt to estimate the relevant point estimates using various imputation methods. The reasons for missing data will be recorded and acknowledged in the manuscript.

### 6.6 Sensitivity analysis

We will conduct a sensitivity analysis to determine the relative weight of constituent studies on the overall point estimate of our review outcome.

### 6.7 Ascertainment of heterogeneity

Q-statistics and *I*^2^ will be used to determine the extent and magnitude of the heterogeneity between selected studies.

### 6.8 Determination of similarity and transitivity

In this review we will assume that the requisite inevitable adjustments between pairwise comparisons will be based on transitivity and similarity.

### 6.9 Assessment of inconsistency

We will determine inconsistency in the network maps by utilizing H^2^ (derivative of the Cochrane Q-statistics) for each point estimate we will compute the corresponding H^2^, and this will serve as a surrogate for the inconsistency per each comparison made.

### 6.10 Ethics and dissemination

The results of this review will be discussed in international conferences, and will be published in a peer-reviewed journal. No formal ethical approval was needed for the purpose of our review, given that it is a secondary synthesis publicly of available literature.

## 7 Discussion

In this systematic review, we will utilize the generalized pairwise modeling, a novel network meta-analytical methodology to generate direct and indirect comparisons between DOAC analogues. We will explore the efficacy and the safety of these agents and attempt to rank them in terms of both their efficacy and safety profile. We think that the results of our review will be important to front line clinicians in informed decisions making about the choice of specific DOAC in patients with CAT. It will also aid guideline and policy makers in the development of therapeutic guidelines in this growing field of medicine.

## Author contributions

Dr. Mohammed Danjuma: review conceptualization, protocol registration, data collection, independent reviewer, risk of bias assessment, and writing the initial draft/revision of the manuscript

Dr Mouhand Faisal Mohamed: Review conceptualization, data analysis, writing the draft and revision of the final manuscript

Dr. Anand Bhaskaran Kartha: review conceptualization Data Collection and writing of initial draft of manuscript

Dr. Mohamed Elshafei: review conceptualization, data collection and cleaning

Dr. Shaikha Al Shokri: data collection, manuscript writing

Dr. Haajra Fatima: data collection, cleaning, and writing of manuscript initial draft

Dr. Sara F H Mohamed: Data collection and revision of the final manuscript

Dr. Mohamed Gaafar Hussein Mohamedali: Provision of expert opinion regarding review and manuscript review

Dr Yahya Mahgoub: review conceptualization, protocol registration, revision

Prof. Abdul-Nasser El-Zouki: Review conceptualization and guidance

Dr. Mubarak Bidmos: Conceptualization, data collection, Risk of Bias assessment, writing the first draft and revision of the final manuscript

Mohammed ibn-mas’ud Danjuma orcid: 0000-0003-2198-5278.
